# Home blood pressure monitoring and equity challenges of the 2025 ACC/AHA hypertension guideline in Africa

**DOI:** 10.11604/pamj.2025.52.12.49067

**Published:** 2025-09-10

**Authors:** Kirubel Tesfaye Hailu, Yeabsera Mekonnen Duguma

**Affiliations:** 1 School of Public Health, University College Cork, Cork, Ireland,; 2 Kadisco General Hospital, Addis Ababa, Ethiopia

**Keywords:** Hypertension, health systems, public health

## Abstract

The 2025 ACC/AHA hypertension guideline reaffirms < 130/80 mmHg as the treatment target and elevates home blood pressure monitoring (HBPM) as the diagnostic gold standard. While this represents progress in hypertension management, it risks widening inequities in Africa, where validated HBPM devices are scarce, unaffordable, and largely excluded from public health systems. Fewer than 10% of households in sub-Saharan Africa own a validated monitor, compared with more than 60% in high-income countries, and retail prices often exceed a week's income. Without deliberate policy action, accurate diagnosis and follow-up may become privileges reserved for wealthier populations, while most patients continue to rely on single clinic readings. Evidence from Ghana, Nigeria, Kenya, and Ethiopia highlights systemic barriers, including device shortages, weak procurement systems, financing gaps, and limited awareness, that undermine hypertension care. To align with international standards, African health systems must recognize HBPM as an essential health technology, add validated devices to national essential lists, and mobilize sustainable financing for procurement. Regional pooled purchasing, local manufacturing, and donor support could reduce costs and improve supply security. Embedding HBPM into hypertension pathways is not only a technical priority but also an equity imperative to close the global treatment gap.

## Commentary

The 2025 ACC/AHA hypertension guideline reaffirms < 130/80 mmHg as the treatment target and elevates out-of-office blood pressure measurement via home blood pressure monitoring (HBPM) or ambulatory monitoring as the diagnostic gold standard [1]. Yet in many low- and middle-income countries, especially across Africa, this evidence-based standard exposes a stark equity gap: validated HBPM devices remain scarce, unaffordable, and absent from most public health systems [2,3].

Surveys show that fewer than 1 in 10 households in sub-Saharan Africa own a validated blood pressure monitor, compared with more than 60% in high-income countries [4]. Many public clinics lack even a single device for patient loan schemes, and HBPM is rarely included on national essential medicines or device lists. Retail prices often exceed US$30-50 per unit, roughly a week's income for many families [3]. Without urgent intervention, the 2025 guideline could unintentionally widen inequities. Accurate diagnosis and follow-up may become a privilege reserved for those able to purchase private devices.

The WHO HEARTS technical package underscores the importance of standardized blood pressure measurement and endorses HBPM where feasible [2], but adoption has lagged due to financing gaps and weak supply chains. Some valuable lessons drawn from the pooled procurement of medicines and vaccines include bulk purchasing of validated monitors, regional tenders, and local manufacturing, which could dramatically lower costs.

Evidence from Ghana highlights that systemic barriers, including device shortages, financing gaps, and distribution challenges, remain significant obstacles to effective hypertension care [3]. Similar challenges recur across the continent. In Nigeria, many primary healthcare facilities lack validated blood pressure monitors, undermining guideline implementation [5]. In Kenya, studies show that up to 80% of individuals with hypertension in urban slums remain undiagnosed, with only about one-third ever screened and less than a quarter on treatment [6]. In Ethiopia, although trained health extension workers can accurately identify hypertension in rural communities, overall knowledge and use of home blood pressure monitoring remains low, even among patients attending public hospitals [7]. These examples underscore that the equity gap is not confined to a single setting but represents a systemic challenge across African health systems.

For African health systems to align with the 2025 ACC/AHA standards, HBPM must be recognized as an essential health technology. Priorities include adding validated devices to national essential lists, mobilizing donor support for procurement, and embedding HBPM within hypertension care pathways. Without these measures, a two-tiered system will persist: accurate, patient-centered care in wealthier settings versus reliance on single clinic readings in resource-limited ones. Equitable access to HBPM is not a technical detail; it is a cornerstone for closing the global hypertension treatment gap [1-3].

## References

[1] Jones DW, Ferdinand KC, Taler SJ, Johnson HM, Shimbo D, Abdalla M (2025). 2025 AHA/ACC/AANP/AAPA/ABC/ACCP/ACPM/AGS/AMA/ASPC/NMA/PCNA/SGIM Guideline for the Prevention. Detection, Evaluation and Management of High Blood Pressure in Adults: A Report of the American College of Cardiology/American Heart Association Joint Committee on Clinical Practice Guidelines. Hypertension.

[2] World Health Organization (WHO) Technical package for cardiovascular disease management in primary health care.

[3] Byiringiro S, Hinneh T, Chepkorir J, Tomiwa T, Commodore-Mensah Y, Marsteller J (2024). Healthcare system barriers and facilitators to hypertension management in Ghana. Ann Glob Health.

[4] Yusuf S, Rangarajan S, Teo K, Islam S, Li W, Liu L (2014). Cardiovascular risk and events in 17 low-, middle-, and high-income countries. N Engl J Med.

[5] Shedul GL, Ripiye N, Jamro EL, Orji IA, Shedul GJ, Ugwuneji EN (2025). Supportive supervision visits in a large community hypertension programme in Nigeria: implementation methods and outcomes. BMJ Open Qual.

[6] Joshi MD, Ayah R, Njau EK, Wanjiru R, Kayima JK, Njeru EK (2014). Prevalence of hypertension and associated cardiovascular risk factors in an urban slum in Nairobi, Kenya: a population-based survey. BMC Public Health.

[7] Teshome DF, Balcha SA, Ayele TA, Atnafu A, Sisay M, Asfaw MG (2022). Trained health extension workers correctly identify high blood pressure in rural districts of northwest Ethiopia: a diagnostic accuracy study. BMC Health Services Research.

